# The impact of the coronavirus disease 2019 pandemic on the clinical presentation of tubal ectopic pregnancies: a retrospective cohort study

**DOI:** 10.1590/1806-9282.20231445

**Published:** 2024-05-17

**Authors:** Onur Yavuz, Sefa Kurt, Mehmet Eyüphan Özgözen, Aslı Akdöner

**Affiliations:** 1 Dokuz Eylül University, School of Medicine, Department of Obstetrics and Gynecology – İzmir, Turkey.; 2 Dokuz Eylül University Hospital – İzmir, Turkey.

**Keywords:** COVID-19, Ectopic pregnancy, Pandemic

## Abstract

**OBJECTIVE:**

We aimed to assess the impact of the coronavirus disease 2019 pandemic on the clinical presentation of tubal ectopic pregnancies.

**METHODS:**

This retrospective cohort study was conducted at a tertiary center and included 76 cases of tubal ectopic pregnancies. The study period was divided into two groups: the pre-coronavirus disease group (January 2018 to February 2020, Group 1; n=47, 61.8%) and the coronavirus disease group (March 2020 to February 2022, Group 2; n=29, 38.2%). Subgroup analysis was also performed for tubal ruptured ectopic pregnancies as Group 1 (n=15, 62.5%) and Group 2 (n=9, 37.5%).

**RESULTS:**

No statistically significant differences were observed between the pre-coronavirus disease and coronavirus disease groups in terms of demographic characteristics. Although the serum beta-human chorionic gonadotropin level was found to be higher in Group 2, the difference was not statistically significant (p=0.7). The groups appeared to be similar in treatment management, duration of hospitalization, and blood transfusion needs (p=0.3, p=0.6, and p=0.5, respectively). Additionally, no significant difference was observed between the groups in the evaluation of ruptured ectopic pregnancies (p=0.5). In the subgroup analysis of tubal ruptured ectopic pregnancies, no significant difference was observed.

**CONCLUSION:**

To the best of our knowledge, there are few studies evaluating the effect of the pandemic on tubal ectopic pregnancies in the literature. Although we did not report statistically significant differences between groups in our study, given the potential prolonged duration of the pandemic, healthcare professionals should actively prompt their patients to seek necessary medical assistance.

## INTRODUCTION

Ectopic pregnancy (EP) is defined as the occurrence of implantation outside the uterine cavity^1^. EPs account for 1–2% of all pregnancies, with nearly 95% of cases occurring in the fallopian tubes^1,2^. In the first trimester, tubal EPs can be diagnosed using serum beta-human chorionic gonadotropin (β-hCG) levels and transvaginal ultrasonography, allowing the detection of about 85% of asymptomatic cases^2^. Early gestational week tubal EPs are often managed with the methotrexate (MTX) regimen or expectant management. However, later gestational week tubal EPs require surgical intervention and cannot be treated expectantly or with MTX^3^. Delay or failure to diagnose tubal EPs promptly can lead to gynecological emergencies with abnormal vital signs, hypovolemic shock, and maternal hemorrhage^4^. Tubal ruptured ectopic pregnancies (rEPs) require immediate surgical intervention and account for about 15% of all EP cases^3^. Tubal rEPs are responsible for three-quarters of maternal deaths in the first trimester. Unfortunately, they are also accountable for about one-sixth of all pregnancy-related deaths^5^. Current surgical indications for EPs include failed MTX treatment, EPs with embryonic cardiac motion (ECM) on ultrasonography, recurrent EP in the same tube, and contraindications to medical or expectant treatment^4^.

In late 2019, the coronavirus disease 2019 (COVID-19) pandemic originated in China and subsequently spread globally. In our country, the first case was reported in March 2020, leading to various restrictions to control transmission. However, these restrictions did not apply to gynecological, obstetric, or other medical emergencies. Despite this, some patients avoided seeking medical care during the pandemic due to concerns about COVID-19 transmission. Consequently, non-COVID-19 medical visits to hospitals significantly decreased during this period^6^. The incidence of molar pregnancy and the number of urgent surgical interventions for tubal rEP have increased during the pandemic^6,7^. This increase is thought to be due to delayed diagnosis. There are few studies evaluating the effect of the pandemic on tubal EPs in the literature^7-9^. However, there is no study in the literature evaluating the effect of the pandemic on tubal rEPs. In this study, first, we aimed to assess the impact of the COVID-19 pandemic on the clinical presentation of tubal EPs. Second, our objective was to evaluate tubal rEPs.

## METHODS

This was a retrospective cohort study conducted at a tertiary center. Institutional ethical approval was provided. Patients had signed informed consent forms, permitting their medical data to be utilized for scientific research, provided that their personal identifiers remain confidential. The study was conducted in accordance with the Helsinki Declaration Principles. Between January 2018 and February 2022, 85 EP patients were diagnosed, followed up, and treated in our clinic. Nine nontubal location EPs (pregnancy of unknown location; n=4, cervical; n=1, cesarean scar; n=1, cornual; n=3) were excluded from the study. The remaining tubal EPs (n=76) were included in the study. March 11, 2020, when the first case of COVID-19 was identified in our country, served as the dividing point between the groups. The cases were classified as pre-COVID (between January 2018 and February 2020, Group 1) and COVID (between March 2020 and February 2022, Group 2). Initially, tubal EPs, followed by tubal rEPs, were analyzed between the groups. The diagnosis and treatment of EPs were managed in accordance with the current literature^10^. The treatment protocol of our institute is as follows: (1) expectant management: stable vital signs, initial serum β-hCG <1,000 IU/L, and 15–20% decrease within 48 h; (2) MTX treatment: stable vital signs, serum β-hCG <5,000 IU/L, non-severe symptoms, and tubal mass size <35 mm, received a single IM dose (50 mg/m^2^) of MTX on day 1. On day 7, if serum β-hCG level dropped ≥15% from day 4, the protocol was ceased. Additional MTX doses were administered if needed. (3) Elective surgical procedure: stable vital signs, serum β-hCG >5,000 IU/L, and ECM or tubal mass size ≥35 mm. (4) Urgent surgical procedure: abnormal vital signs, severe symptoms, and significant free fluid on ultrasonography.

Demographic characteristics, laboratory data, ultrasonographic findings, clinical symptoms, treatment methods, intraoperative findings, and follow-up data were obtained from hospital records. On the ultrasonography examination, the amount of free fluid filling the pelvis was accepted as increased. Intraoperative increased intrabdominal free fluid was defined as hemoperitoneum >1,000 mL. A drop in hemoglobin >2 g/dL or abnormal vital signs necessitated a blood transfusion.

Analyses were performed with SPSS version 26.0 (IBM Inc., Chicago, IL, USA). Normality analysis was performed according to the Kolmogorov-Smirnov test. Non-normally distributed parameters were analyzed with the Mann-Whitney U test. Quantitative data were presented as median (minimum–maximum), and qualitative data were presented as numbers and percentages (%). Chi-square and Fisher precision tests were used for the analysis of categorical data. The p-value considered statistically significant was <0.05.

## RESULTS

Between January 2018 and February 2022, 85 EPs were diagnosed, followed up, and treated in our clinic. Nine nontubal location EPs (pregnancy of unknown location; n=4, cervical; n=1, cesarean scar; n=1, cornual; n=3) were excluded from the study. These nine EPs were in the COVID period group. The remaining tubal EPs (n=76) were analyzed. Between January 2018 and February 2022, 89.4% (76/85) of all EPs were tubal EPs. The rate of tubal EPs was 100% (47/47) in the pre-COVID period and 76% (29/38) in the COVID period. The cases were classified as pre-COVID (Group 1; n=47, 61.8%) and COVID (Group 2; n=29, 38.2%). Then, tubal rEPs (n=24) were analyzed between the groups. Tubal rEPs accounted for 31.5% (24/76) of the study cohort.

Table 1 presents the demographic characteristics of the study groups. The median age of study participants was 32.5 years. The median age of Group 1 was 32 years and Group 2 was 34 years (p=0.9). The median gravida and parity of the study participants were 2 and 1, respectively. The median gravida of Group 1 was 2 and Group 2 was 2 (p=0.9). The median parity of Group 1 was 0 and Group 2 was 1 (p=0.2). The groups did not exhibit a statistically significant difference in terms of abortion rates (48.9 vs. 34.5%, p=0.2). There was no significant association between the groups regarding previous EP occurrences (19.1 vs. 10.3%, p=0.2). The incidence of previous cesarean section, intrauterine device use, and smoking habit did not exhibit statistically significant differences between the two groups. No patient has received oral contraceptive pill medication. Although the previous gynecological operation rate of Group 2 was higher, this difference was not statistically significant (10.6 vs. 27.6%, p=0.05). There was no significant relationship between the groups in terms of either assisted conception or previous ultrasound (8.5 vs. 17.2%, p=0.2 and 70.2 vs. 69%, p=0.5, respectively). Both groups had statistically similar median gestational ages (6 vs. 6, p=0.4).

**Table 1:** Demographic characteristics of groups.^a,b^

	Group 1 n=47 (61.8%)	Group 2 n=29 (38.2%)	All cases n=76 (100%)	p-value
Maternal age (years)	32 (22–41)	34 (21–45)	32.5 (21–45)	0.9
Gravida	2 (1–10)	2 (1–6)	2 (1–10)	0.9
Parity	0 (0–3)	1 (0–4)	1 (0–4)	0.2
Previous abortion	23/47 (48.9%)	10/29 (34.5%)	33/76 (43.4%)	0.2
Previous ectopic pregnancy	9/47 (19.1%)	3/29 (10.3%)	12/76 (15.8%)	0.2
Previous C/S	13/47 (27.7%)	5/29 (17.2%)	18/76 (23.7%)	0.2
IUD	1/47 (2.1%)	0/29 (0%)	1/76 (1.3%)	0.6
OCP	0/47 (0%)	0/29 (0%)	0/76 (0%)	–
Smoking habits	4/47 (8.5%)	1/29 (3.4%)	5/76 (5.5%)	0.3
Previous gynecological operation	5/47 (10.6%)	8/29 (27.6%)	13/76 (17.1%)	0.05
Assisted reproduction	4/47 (8.5%)	5/29 (17.2%)	9/76 (11.8%)	0.2
Gestation age^c^	6 (4–9)	6 (5–9)	6 (4–9)	0.4
Prior ultrasound	33/47 (70.2%)	20/29 (69%)	53/76 (69.7%)	0.5

C/S: cesarean section, IUD: intrauterine device, OCP: oral contraceptive pills.

^a^Values are given as numbers (percentage, %) unless stated otherwise.

^b^Values are given as median (minimum–maximum) unless stated otherwise.

^c^Based on menstrual dates.

The clinical, laboratory, and ultrasonography findings of the groups are listed in Table 2. The groups were statistically similar in terms of abdominal pain and vaginal bleeding (76.6 vs. 58.6%, p=0.08 and 85.1 vs. 72.4%, p=0.1, respectively). The serum β-hCG level of Group 2 was found to be higher (2,261 vs. 2,450 IU/mL, p=0.7). The levels of hemoglobin were similar in both groups (11.9 vs. 11.8 g/dL, p=0.7). Regarding ECM, it was detected in 8.5% of the patients in Group 1, while no ECM was detected in Group 2 (p=0.1). The presence of increased intraabdominal free fluid showed no statistically significant difference between the two groups (31.9 vs. 31%, p=0.5). In the evaluation of the rEPs, no differences were observed between the groups (31.9 vs. 31%, p=0.5). The treatment management was statistically similar between the two groups (p=0.3). Expectant treatment is more prevalent in Group 2 (8.5 vs. 13.5%, p=0.4). MTX and additional MTX dose requirements are higher in Group 1 (40.4 vs. 31%; p=0.4 and 10.6 vs. 10.3%; p=0.9, respectively). Laparoscopic salpingectomy was higher in Group 2, while laparatomic salpingectomy was higher in Group 1 (25.5 vs. 41.4%; p=0.1 and 14.9 vs. 3.4%; p=0.1, respectively). MTX failure was detected in one patient in Group 1 and two patients in Group 2 (5.3 vs. 22.2%; p=0.1). In these cases, tubal rupture occurred and urgent laparoscopic salpingectomy was performed. The groups showed no statistically significant difference in terms of hospitalization (2.2 vs. 2.6%, p=0.6) and the need for blood transfusion (14.9 vs. 17.2%, p=0.5).

**Table 2:** Clinical, laboratory, and ultrasonography findings of groups.^a,b^

	Group 1 n=47 (61.8%)	Group 2 n=29 (38.2%)	All cases n=76 (100%)	p-value
A) Clinical findings				
Abdominal pain	36/47 (76.6%)	17/29 (58.6%)	53/76 (69.7%)	0.08
Vaginal bleeding	40/47 (85.1%)	21/29 (72.4%)	61/76 (80.3%)	0.1
B) Laboratory at presentation				
β-hCG level (IU/mL)	2,261 (57–53,000)	2,450 (75–26,980)	2,301 (57–53,000)	0.7
Hemoglobin (g/dL)	11.9 (8.1–14.4)	11.8 (9.4–14.6)	11.8 (8.1–14.6)	0.7
C) Ultrasonography				
Embryonic cardiac motion	4/47 (8.5%)	0/29 (0%)	4/76 (5.3%)	0.1
Increased intraabdominal free fluid^c^	15/47 (31.9%)	9/29 (31%)	24/76 (31.6%)	0.5
D) Ruptured ectopic pregnancy	15/47 (31.9%)	9/29 (31%)	24/76 (31.6%)	0.5
E) Treatment management				0.3
Expectant	4/47 (8.5%)	4/29 (13.5%)	8/76 (10.5%)	0.4
Methotrexate	19/47 (40.4%)	9/29 (31%)	28/76 (36.8%)	0.4
Additional methotrexate dose requirements	5/47 (10.6%)	3/29 (10.3%)	8/76 (10.5%)	0.9
Laparoscopic salpingectomy	12/47 (25.5%)	12/29 (41.4%)	24/76 (31.6%)	0.1
Laparatomic salpingectomy	7/47 (14.9%)	1/29 (3.4%)	8/76 (10.5%)	0.1
Methotrexate failure	1/19 (5.3%)	2/9 (22.5%)	3/28 (10.7%)	0.1
F) Hospitalization (days)	2 (1–7)	1 (1–14)	2 (1–14)	0.6
G) Blood transfusion needs	7/47 (14.9%)	5/29 (17.2%)	12/76 (15.8%)	0.5

^a^Values are given as numbers (percentage, %) unless stated otherwise.

^b^Values are given as median (minimum–maximum) unless stated otherwise.

^c^Estimated by the physician during the ultrasound examination.

Table 3 shows the comparison of the groups who underwent urgent surgery for tubal rEPs. Laparoscopic salpingectomy was performed in eight patients (53.3%) in Group 1 and seven patients (89%) in Group 2. Laparatomic salpingectomy was performed in seven patients (46.7%) in Group 1 and one patient (11%) in Group 2. The difference in surgical management between the groups was not statistically significant (p=0.08). The observed difference in serum β-hCG levels between Group 1 and Group 2 was not statistically significant (3,621 vs. 8,888 IU/mL, p=0.2). Higher preoperative hemoglobin levels were observed in Group 2 (11.8 vs. 12.6 g/dL, p=0.2), while postoperative hemoglobin levels were lower in Group 1 (8.3 vs. 9 g/dL, p=0.2). The postoperatively evaluated hemoglobin decrease was higher in Group 2 (1.3 vs. 2.8 g/dL, p=0.08). Increased abdominal free fluid on ultrasonography and intraoperative free fluid was detected in all cases of both groups. The groups were statistically similar in terms of hospitalization and blood transfusion need (3 vs. 3, p=0.8; 46.6 vs. 44.4%, p=0.6, respectively).

**Table 3:** Comparison of groups who underwent urgent surgery for ruptured tubal ectopic pregnancies.^a,b^

	Group 1 n=15 (62.5%)	Group 2 n=9 (37.5%)	All cases n=24 (100%)	p-value
A) Surgery management				0.08
Laparoscopic salpingectomy	8/15 (53.3%)	8/9 (89%)	16/24 (66.6%)	
Laparatomic salpingectomy	7/15 (46.7%)	1/9 (11%)	8/24 (33.3%)	
B) Laboratory findings				
α - hCG level (IU/mL)	3,621 (57–53,000)	8,888 (152–26,980)	4,186 (57–53,000)	0.2
Preoperative hemoglobin (g/dL)	11.8 (8.1–13.4)	12.6 (9.4–13.2)	12 (8.1–13.4)	0.2
Postoperative hemoglobin (g/dL)	8.3 (6.1–12.2)	9 (6.6–12.1)	8.7 (6.1–12.2)	0.2
Hemoglobin drop (g/dL)	1.3 (0.6–5.7)	2.8 (0.6–5.7)	2.1 (0.6–5.7)	0.08
C) Ultrasonography findings				
Increased abdominal free fluid^c^	15/15 (100%)	9/9 (100%)	24/24 (100%)	–
D) Intraoperative free fluid^d^	15/15 (100%)	9/9 (100%)	24/24 (100%)	–
E) Hospitalization (days)	3 (2–4)	3 (2–14)	3 (2–14)	0.8
F) Blood transfusion needs	7/15 (46.6%)	4/9 (44.4%)	11/24 (45.8)	0.6

^a^Values are given as numbers (percentage, %) unless stated otherwise.

^b^Values are given as median (minimum–maximum) unless stated otherwise.

^c^Estimated by the physician during the ultrasound examination.

^d^Intraoperative observation by the surgeon of free fluid filling at least the Douglas pouch.

## DISCUSSION

In this retrospective cohort study, our primary objective was to assess the impact of the COVID-19 pandemic on the clinical presentation of tubal EPs. The secondary objective was to evaluate tubal rEPs. The study findings revealed that the pandemic did not have a statistically significant effect on the management and incidence of tubal EPs and rEPs.

Approximately 95% of EPs are localized in the tuba uterina^3^. In our study, between January 2018 and February 2022, 89.4% of all EPs were tubal. Aiob et al. reported tubal EPs were 89.8% of all EPs in the pre-COVID period^8^. This incidence was 89.4% in the COVID period^8^. We found that the rate of tubal EPs was 100% in the pre-COVID period and 76% in the COVID period. These results align with the existing literature and indicate that tubal EPs remain the most common type, with a slight insignificant decrease observed during the COVID period.

In the reviewed studies, no statistically significant differences were observed between the prepandemic and pandemic groups concerning demographic characteristics, including maternal age, gravida, parity, and previous cesarean section^7-9^. Our study findings were consistent with these reports.

Barg et al. investigated the effect of the pandemic on EPs^7^. They have found no difference between the groups in gestational week^7^. On the contrary, Aiob et al. reported that the gestational week of EPs was approximately 1 week higher in the pandemic period^8^. This difference was statistically significant. In our study, the gestational weeks of both groups were similar.

Throughout our research, we observed a relatively higher level of serum β-hCG at the initial visit in Group 2. Nevertheless, it is noteworthy to mention that this disparity was not statistically significant. These findings are in accordance with other studies that have also reported higher serum β-hCG levels in EPs during the pandemic period^7-9^. Furthermore, Barg et al. made a noteworthy observation regarding the serum β-hCG levels in the COVID group, which were found to be twice as high as those in the pre-COVID group^7^. The researchers reported that this difference was statistically significant.

In their study, Aiob et al. compared the complaints of EPs between the prepandemic and pandemic periods^8^. They found that abdominal pain exhibited a statistically significant difference between the prepandemic and pandemic groups, with the pandemic group having a 1.6 times higher prevalence of abdominal pain. Similarly, the pandemic group showed a higher rate of vaginal bleeding symptoms. However, this finding was not statistically significant. On the contrary, our study yielded different results, indicating that these symptoms were actually lower in the COVID group. Despite these differences in symptom prevalence, the overall comparison between the two groups showed no statistically significant differences, suggesting that the COVID period did not significantly influence the occurrence of these specific symptoms in our cohort of patients with tubal EPs.

Barg et al. have revealed notable differences in the management and outcomes of tubal EPs between the pre-COVID and COVID periods^7^. Expectant management was twofold more common in the pre-COVID period, whereas MTX treatment was administered at similar rates between the groups. However, MTX failure was threefold higher in the COVID group. Additionally, the COVID group showed a 1.3-fold increase in the number of elective and urgent laparoscopic surgeries. Tubal rEPs were also three times more common in the COVID group. All of these differences in treatment approaches and surgical interventions were statistically significant.

Aiob et al. reported that there was no statistically significant difference between the groups concerning expectant and MTX management^8^. This indicates that both the pandemic and prepandemic groups received similar treatment approaches for their condition, with no significant variation in management strategies between the two groups according to their study findings^8^. Although ruptured EPs were more common in the pandemic group, they occurred at a lower rate. Nonsurgical management was similar in both groups. In the pandemic group, the count of urgent laparoscopies was found to be twice as high as that in the prepandemic group. Conversely, the prepandemic group had three times more elective laparoscopies than the pandemic group. These findings indicate a notable difference in the urgency and timing of laparoscopic procedures between the two groups during the study period. These differences were found to be statistically significant. In our study, we did not observe any significant difference between the groups in the evaluation of rEPs. The findings indicate that both groups showed similar rEP patterns, suggesting no distinct impact of the COVID period on rEPs in comparison with the pre-COVID period. Additionally, the treatment management was similar between the two groups. Expectant management was higher in Group 2. MTX treatment and additional MTX dose requirements were higher in Group 1. Laparoscopic salpingectomy was higher in Group 2, while laparatomic salpingectomy was higher in Group 1. MTX failure was 4.2 times more likely in Group 2, which was higher than the rate reported in the literature. However, the difference was not statistically significant. In these cases, tubal rupture had occurred and urgent laparoscopic salpingectomy was performed. These findings highlight the importance of appropriate and timely interventions in managing tubal EPs, particularly when faced with potential treatment challenges during the COVID period. Dvash et al. have reported that tubal rEPs were twofold higher in the pandemic group^9^. This difference was statistically significant. In their analysis, the researchers found that no rEPs were detected in women who became pregnant after undergoing assisted reproduction. The researchers proposed another factor contributing to the absence of rupture in such patients: frequent medical observation and high patient awareness. This vigilance and proactive approach facilitated early detection and streamlined treatment protocols, potentially mitigating the risk of rupture and improving patient outcomes. Serum β-hCG levels of the pandemic group were twice that of the prepandemic group. However, there was no statistically significant difference between the groups. Both groups were similar in terms of non-surgical and surgical management. Interestingly, the duration of hospitalization was longer and the amount of intraabdominal free fluid observed preoperatively was higher in the prepandemic group. In our study, the incidences of rEP were similar between groups. Although serum β-hCG levels were higher in Group 2, there was no statistical difference between the groups. Both groups were similar in terms of assisted reproduction. The tubal rupture status of these patients was not evaluated separately. The duration of hospitalization was longer in Group 2. Group 2 had higher preoperative hemoglobin levels and lower postoperative hemoglobin levels. Postoperatively evaluated hemoglobin drop was higher in Group 2. The groups were similar in terms of blood transfusion needs. However, these data were not statistically different. As reported in tubal rEP analysis, laparoscopic salpingectomy was performed in eight patients in Group 1 and seven patients in Group 2. Laparatomic salpingectomy was performed in seven patients in Group 1 and one patient in Group 2. There was no statistically significant difference between the groups in terms of surgical management. In our clinic, we prefer the laparoscopic method as the current approach. Exceptionally, hemodynamically unstable patients with diffuse intraabdominal free fluid on ultrasonography may undergo urgent laparotomy.

The retrospective design is a limitation of our study. Vaccinations during the pandemic period were not examined. Another limitation of our study is the small population size. The strength of our study is that it includes data from a single-center tertiary hospital. In addition, especially during the pandemic period, our clinic served as a reference center.

In conclusion, to the best of our knowledge, there are few studies evaluating the effect of the pandemic on tubal EPs in the literature. Unlike other studies, we performed a detailed subgroup analysis specifically focusing on tubal rEPs. Performing a detailed subgroup analysis, specifically focusing on tubal rEPs, can indeed yield more precise and valuable insights into the impact of the pandemic on this specific subgroup of patients.
